# A Survey on Modelling of Automotive Radar Sensors for Virtual Test and Validation of Automated Driving

**DOI:** 10.3390/s22155693

**Published:** 2022-07-29

**Authors:** Zoltan Ferenc Magosi, Hexuan Li, Philipp Rosenberger, Li Wan, Arno Eichberger

**Affiliations:** 1 Institute of Automotive Engineering, Graz University of Technology, 8010 Graz, Austria; hexuan.li@tugraz.at (H.L.); arno.eichberger@tugraz.at (A.E.); 2 Institute of Automotive Engineering, Technical University Darmstadt, 64289 Darmstadt, Germany; philipp.rosenberger@tu-darmstadt.de; 3 IPG Automotive GmbH, 76185 Karlsruhe, Germany; li.wan@ipg-automotive.com

**Keywords:** radar sensor, machine perception, radar sensor model, automated driving, virtual testing

## Abstract

Radar sensors were among the first perceptual sensors used for automated driving. Although several other technologies such as lidar, camera, and ultrasonic sensors are available, radar sensors have maintained and will continue to maintain their importance due to their reliability in adverse weather conditions. Virtual methods are being developed for verification and validation of automated driving functions to reduce the time and cost of testing. Due to the complexity of modelling high-frequency wave propagation and signal processing and perception algorithms, sensor models that seek a high degree of accuracy are challenging to simulate. Therefore, a variety of different modelling approaches have been presented in the last two decades. This paper comprehensively summarises the heterogeneous state of the art in radar sensor modelling. Instead of a technology-oriented classification as introduced in previous review articles, we present a classification of how these models can be used in vehicle development by using the V-model originating from software development. Sensor models are divided into operational, functional, technical, and individual models. The application and usability of these models along the development process are summarised in a comprehensive tabular overview, which is intended to support future research and development at the vehicle level and will be continuously updated.

## 1. Introduction

It has already been shown that supporting the human driver in complex traffic situations and transferring some or all of the driving tasks to automated driving systems increases traffic safety, efficiency of energy use and traffic flow, and travel comfort. Technological advances in semiconductors and information technology are enabling the development of increasingly sophisticated sensors, decision algorithms, and intervention elements. The technology required for automated driving has now advanced to the point where thousands of kilometres can be travelled accident-free. Given the economic and societal benefits of automated driving systems (ADS) and the level of technological development required for their deployment, rapid market penetration would be warranted. Since the development of motor vehicles and the market introduction of their functions are subject to stringent legislation and validation measures, automotive manufacturers must ensure that automated driving functions (ADF) provide more safety than the human driver. In the automotive industry, testing and validation methods are used to ensure the desired level of safety for less automated systems through the reliability of software and hardware components. With the introduction of driving automation systems (DAS) and automated driving systems, referred to as DAS/ADS systems, vehicles are becoming more complex, so the test and validation tasks go far beyond reliability testing of the vehicle’s hardware and software components.

The methodology currently used in the automotive industry follows the V-model development process. In this approach, system requirements are defined in parallel with their verification and validation (V&V) activities throughout the development process. The concept of V&V is to find an appropriate way to determine whether a product, function, system, or subsystem meets or complies with safety requirements, specifications, and regulations. A comprehensive V&V methodology is required to ensure that the complex vehicle system will operate safely in an unsafe traffic environment. As system complexity increases, traditional V&V methodologies, including on-road and test-bed testing, are no longer sufficient to meet safety requirements through meaningful coverage of test cases and scenarios. To close this gap, simulation must go beyond development support and become a credible test tool for virtual approval and homologation of safety-critical ADS.

DAS/ADS systems combine capabilities that fit into one of the three segments of the sense-plan-act model adopted from the robotics and automation literature, and this model provides a high-level, implementation-independent view of a complex automated vehicle system.

The testing and validation activities of the automotive industry are closely linked to the regulatory framework. Product validation and quality assessment are continuously performed according to industry-specific standards. Following the ISO-26262 standard (ISO-International Organization for Standardization), one of the most important safety standards in the automotive industry, conformity assessment is carried out through comprehensive test procedures. The traditional test procedures of model-in-the-loop (MiL), software-in-the-loop (SiL), processor-in-the-loop (PiL), hardware-in-the-loop (HiL), driver-in-the-loop (DiL), and vehicle-in-the-loop (ViL), also referred to as X-in-the-loop, have led to detailed testing and modelling of the integrated vehicle dynamics as well as the entire vehicle system, providing well-developed models for testing the plan and act capabilities in the framework. Sensor models have also been developed to improve DAS/ADS capabilities and are used in state-of-the-art (SOA) environmental simulation software. However, there is still a significant discrepancy between the results of real sensors and the results of most sensor models in terms of all possible effects that occur during signal propagation and processing, such as due to tire spray or multipath propagation. However, it should be noted at this point that in order to perform simulations efficiently, models with varying degrees of realistic representation must be used, depending on the stage of development and the specific scenario.

In our review, we focus on radio detection and ranging (radar) sensor models because they have special characteristics due to the complexity of radar wave propagation. This complexity has led to a variety of proposed simulation approaches.

The remainder of the paper is structured as follows: Section 2 summarises challenges in automotive testing procedures, Section 3 presents the vehicle development process and the usage of sensor models, Section 4 gives an overview of previous survey in radar sensor modelling, Section 5 introduces a new classification approach that is more useful for selection of the appropriate modelling approach, and Section 6 discusses the findings of the paper.

## 2. The Challenge in Automotive Testing

**Figure 1:**
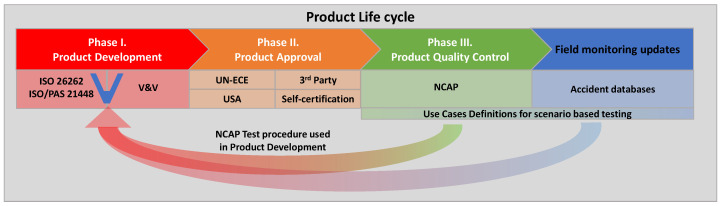
Automotive product life cycle phases related to V&V of safety.

In recent years, more and more advanced features have been incorporated into vehicles. The robustness and reliability of these systems is highly dependent on their sensing capabilities, their processing of complex perception algorithms, and their operation by electrical and/or electronic (E/E) systems. The testing and validation activities for such distributed systems are already a complex task and are closely related to various national and international laws, regulations, and industry standards. Szalay et al. identify in [1] three different areas of automotive testing activities according to the life cycle management process of automotive products. Figure 1 illustrates these different phases of vehicle development, which will be explained in the next section. We therefore assume that the modelling approaches for radar sensors must differ in the different product development phases.

### 2.1. Vehicle Development Phase

In the first phase of the product life cycle, manufacturers are responsible for developing safe, reliable, and well-functioning vehicles. The basic legal framework for the development of vehicles or safety-critical systems is set out in product liability legislation. Product liability requires that the product placed on the market must provide a reasonable expectation of safety and must be developed according to the state of the art. These methods are specified in national and international standards maintained by the International Organization for Standardization and/or national standards bodies. The main objective of standardisation work is to ensure the comparability and consistency of analysis results performed independently by different companies [2]. In the automotive industry, development processes are carried out according to the V-model proposed in ISO-26262 [3]. This approach defines system requirements in parallel with their verification and validation throughout the development process, including the software and hardware development phases with the corresponding test activities, especially the various X-in-the-loop test solutions. Standardised test and validation methods are only available for DAS functions with lower levels of automation (SAE level L0-L2).

Requirements are usually set at the vehicle level; for longitudinal control, these are defined for adaptive cruise control (ACC) in ISO-15622 [4] and for autonomous emergency braking (AEB) in ISO-22733 [5]. For lateral guidance, there are standards for lane departure warning (LDW) systems in ISO-17361 [6], for lane change decision aids systems (LCDAS) in ISO-17387 [7], for partially automated lane change systems (PALS) in ISO-21202 [8], and for lane keeping assistance systems (LKAS) in ISO-11270 [9].

### 2.2. Type Approval Phase

In the second phase of the product life cycle, vehicles are brought to market. If an automobile manufacturer wishes to market its product in Europe, it must meet the requirements of the United Nations Economic Commission for Europe (UN-ECE) in a process known as type approval (or homologation). In type approval, vehicles are tested by an independent third party (e.g., TÜV, Dekra), and approval is granted by the authorities. In contrast, there is also the process of self-certification (e.g., in the U.S.), in which the vehicle manufacturer analyses and certifies the safety strategy through a voluntary safety self-assessment to confirm that the product meets market requirements [1]. The criticality of these testing activities is much higher than for feature development. The reason is that if type approval/homologation is successful, the vehicle is deemed safe by the authority and therefore must meet the authority’s specifications and the public’s expectations [2]. For longitudinal control, type approval is specified in UN-ECE R 131 for the advanced emergency braking system (AEBS) [10] and for lateral control in UN-ECE R 157 [11] for the automated lane keeping system (ALKS).

### 2.3. Consumer Protection Phase

In the third phase of the product life cycle, products are already on the market and consumer organizations must demonstrate the quality of the product to protect consumers from the risk of unfair commercial interactions. In the automotive industry, the New Car Assessment Program (NCAP) is one of the best-known consumer protection organizations [1]. It was established in the late 1970s by the U.S. National Highway Traffic Safety Administration (NHTSA) and focuses on testing the major operating and safety systems of motor vehicles. Later, NCAPs were adopted by the automotive industry in other parts of the world, such as the European New Car Assessment Program (Euro NCAP), the Japanese New Car Assessment Program (JNCAP), and the Chinese New Car Assessment Program (C-NCAP) [12]. Euro NCAP’s test protocols, developed based on real-world accident scenarios, are not just a test and evaluation procedure for the final product. The automotive industry also uses them as a tool to directly improve safety by incorporating them into the vehicle development process. By virtually performing NCAP test evaluation protocols with the MiL and SiL simulation methods, development engineers can elaborate and verify the vehicle’s perception strategy in a time- and cost-efficient manner at an early stage of development, during the concept phase. They also provide a good basis for functional testing of software and hardware prototypes on HiL test benches in later development phases [13].

## 3. Vehicle Development Process

**Figure 2:**
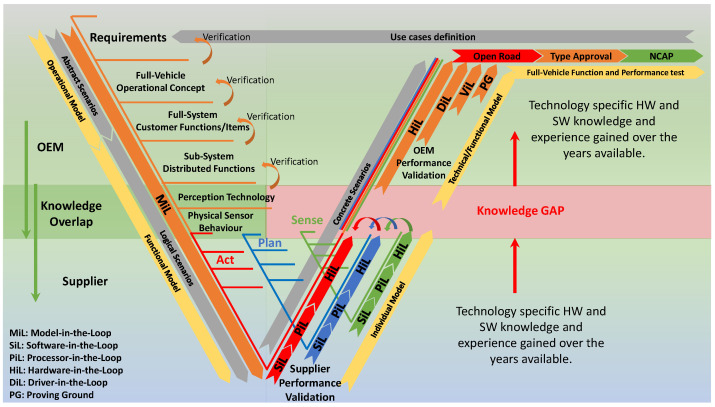
Vehicle development process with respect to driving automation.

The inherent complexity of modern systems is increasing significantly. Consequently, the system development process and the verification and validation process are becoming increasingly complex. In order to cope with this major challenge, suitable development processes have been introduced. Combining these with appropriate simulation techniques, different system designs can be evaluated and the number of physical prototypes can be reduced. The increasing complexity of modern vehicle systems requires a modular system design, both in terms of the integrated hardware components and the algorithms running on them. This means that the operation and correct behaviour of the system depend on the interactions between the modules and the content and quality of their input and output information. Therefore, it is becoming increasingly important to test and evaluate the entire system in its intended operating environment. Traditional system design methods address this complexity problem by providing appropriate process models that contain detailed specifications of all components as well as the overall system with all relevant interfaces and relationships. The product development method currently used in the automotive industry is based on the V-model as proposed in the ISO-26262 standard [3] (see Figure 2).

Product development starts with a hierarchical top-down analysis and design phase, followed by implementation and a reverse bottom-up integration and test phase. Test tools corresponding to the development phases, such as MiL, SiL, PiL and HiL simulations, are also provided. The development of modern systems requires the integration of many disciplines, leading to a need for standardised interfaces and coordination between the standard methods of the disciplines involved [14]. To meet this challenge, the methodology of model-based systems engineering (MBSE) plays an increasingly important role in system design, that is, the formalised application of modelling to support the entire development process at all levels of abstraction [15]. The integration of virtual models into the vehicle development process has led to the systematic use of simulation techniques that enable virtually based V&V to prove the correctness of the system already in the requirements analysis phase. One of the key points of this methodology is the V&V concept, as different models of varying complexity can be used for the V&V activities in the different phases of the product development cycle, depending on their complexity. Once the complete system is designed, specified, and verified, it can be implemented, integrated, and validated. The validation processes are designed to ensure that all requirements arising from the ’safety by engineering’ strategy along the left-hand side of the V-model in a decreasing direction are met, that known scenarios are covered, and that the system behaves as specified. In the validation processes, which represent the right side of the V-model, the verified system is tested using well-defined test methods in an ascending direction to confirm that the system meets all safety design requirements and behaves as intended and specified.

In order to validate the safety of the intended functions and systems, the vehicles are tested in all phases of development using a variety of predefined realistic test scenarios in virtual and real traffic. A number of validation tools are already defined in the standards mentioned above, such as SiL and HiL. At the level of the complete vehicle, real test drives are performed on the proving ground, but also on public roads, to ensure that the systems function properly in road traffic. Combining simulations in the above context with tests at the vehicle level is intended to establish statistical confidence in operational safety. As specified in the ISO/PAS-21448 Safety of the Intended Functionality [16] and ISO-26262 Functional Safety standards, once the ADS is on the market, the safety of the system will be continuously monitored through field operational test (FOT) by collecting and analysing anonymous data from the field during on-road testing.

Figure 2 also illustrates that there is a knowledge gap in V&V of automotive radar sensor based systems in the phase where there is a hand-over from the system supplier to the vehicle’s original equipment manufacturer (OEM). It also includes a proposal of which overall modelling approaches are appropriate in the different phases. These approaches will be explained in Section 5.

## 4. State-of-the-Art Radar Sensor Model Classifications

In recent years, many different approaches to modelling radar sensor systems have been developed. An early example of radar sensor modelling can be found in [17], from 1990. There are a number of different approaches to generating synthetic radar data. The authors in [18] distinguish between ideal models, probabilistic models, and physics-based models. They also distinguish three fidelity levels for sensor models with increasing complexity: low-fidelity models, medium-fidelity models, and high-fidelity models [19]. Ideal sensor models simply generate ground truth (GT) data for objects in the sensor’s detection range. This allows sensor-type-specific object list information to be generated for all objects in the sensor’s field of view (FOV). In contrast to ideal sensor models, physics-based sensor models attempt to model the physical sensing process of the real sensor as accurately as possible. They are computationally intensive and require more computational power, often at the expense of real-time capability. In addition to the required expertise in sensor technology, a detailed description of the environmental conditions (material properties, weather conditions, etc.) is required to accurately model the physics of the sensor. The simulated output data from these models can be a raw analog signal comparable to that of the real sensor. As suggested in [20,21], the use of probabilistic sensor models can provide a reasonable trade-off between complexity and computational efficiency. The simplified parameter set and reduced model complexity allow simulation tests to be performed faster than real time. The output of these models can be set up to provide object lists or even raw data. Even though the output data is less realistic compared to the real sensor, phenomenological models can be used at most levels of the development process to test and validate the safety of the ADS.

Rosenberger et al. in [22] distinguish between ground truth models that neglect any operation on the GT object list except for the transformation from world to sensor origin coordinates, idealised models that additionally cut out the FOV of the sensor from the object list, and phenomenological models that consist of stochastic and physical model parts. In radar modelling, this could mean that a ray tracing approach to physical modelling of signal propagation is accompanied by a stochastic Gaussian noise model. While pure stochastic modelling is often applied in sensor models for high-level object list output, pure physical modelling is almost completely avoided in scenario-based simulation due to the computational time required for such finite element method (FEM) simulation or the like.

In [23], the author categorises sensor models into three groups, defined as ideal, physical, and functional. Ideal sensor models directly read any measurable objects or GT information provided in the virtual environment without including any real sensor-related uncertainty. In contrast, physical models or white-box models implement the real physical sensor properties, but at the expense of real-time simulation performance. The functional model ignores the sensor hardware architecture and signal processing process. Such a black-box model focuses only on the detection result of the measured object. This type of sensor model can be implemented by combining certain geometric and selected physical properties. This improves the real-time performance of the sensor simulation without completely ignoring the detection limits and characteristics of the real sensor.

Similarly, in [24], sensor models are classified as function-based, physics-based, or a combination of both. For simplicity and real-time capability, the function-based modelling approach includes geometric models and models dealing with scattering centres. The physics-based approach has been divided into two classes, one dealing with modelling the electronic components of the radar sensor, including the propagation channel modelled by ray-tracing techniques. The other approach considers radar and clutter echoes as well as noise. Another approach is presented that distinguishes between a radar model and a radar system model, where the radar system model includes the environment and the target vehicle model in addition to the radar model.

Holder et al., in [25], distinguish three groups of sensor models and define them as follows. The ideal sensor models generally generate a list of perfectly sensed objects from the simulation environment, that is, they do not model sensing errors. These are followed by phenomenological sensor models that already take into account additional sensor properties such as the FOV of the sensor, limited resolution, and measurement uncertainties [26,27]. Finally, physical sensor models aim to reproduce the raw sensor data by modelling the physical phenomena specific to the sensor being modelled. The authors in [28,29,30] provide an assessment of radar sensor models in the literature, based on some predefined modelling criteria, with the goal of helping the modeller estimate the effort required to create such models. Based on their defined criteria, radar sensor models can be classified into three categories. In the physical sensor model, called the white-box model, all physical aspects of the radar are considered and calculated based on a detailed description of the environment [24,31,32]. The scattering centre sensor models exploit the property, known from radar cross-section (RCS) studies, that electromagnetic scattering from an electrically large target can be approximated by a sparse set of points at a fixed position on the target, called scattering centres (SC) [33,34,35]. Data-driven or black-box models do not require information about the behaviour of the radar and can only learn its operation based on recorded data from real experiments [29,36,37].

Schlager et al. [19] determine the accuracy of a sensor model based on its inputs, outputs, and modelling principle. Low-fidelity sensor models are based on geometric aspects such as the sensor-specific FOV and object occlusion. The input data format is object lists with ground truth information, and the output data format is also object lists but with filtered GT information [38]. Medium-fidelity sensor models take into account some physical aspects of the real sensor and material properties of the objects, as well as the sensor’s field of view and detection probability. The input for medium-fidelity sensor models are object lists corresponding to ground truth. The output data formats are object lists or raw data processed, according to the modelled perceptual effects [39]. High-fidelity sensor models are the most accurate representations of real-world sensors. They incorporate rendering methods such as rasterisation or ray tracing. They combine environmental parameters and material properties as well as physical effects such as diffraction and interference. High-fidelity sensor models use the entire 3D virtual environment, a mesh grid describing objects and their surfaces, as input and produce sensor-specific raw data as output [40].

Cao et al. [41] introduced the white-box, grey-box, and black-box classifications for sensor models. Black-box models can be used for system function verification but not for validation. White-box modelling is the simulation of electromagnetic (EM) wave propagation by solving Maxwell’s equations, simulating semiconductor components and the propagation channel. Grey-box modelling is an effective combination of the above models in terms of complexity and real-time capability.

Ngo et al. [42] distinguish time-domain electromagnetic simulation techniques, ray tracing, data-driven, and idealised modelling approaches. They describe a method for evaluating a radar sensor model by comparing the results of clustering algorithms with real and synthetic radar data and provide a sensitivity analysis for the various parameters of their radar sensor model.

In a later publication, Ngo et al. [43] describe common sensor model categorisation into physics-based, probabilistic, and phenomenological. They also describe the expansion stages of their radar models, which are later used to demonstrate their multilayer model validation study: ideal radar model (IRM), data-driven model (DDM), and ray-tracing-based model (RTM).

Holder in [44] classifies sensor models according to the information flow in the simulation, the model input (I) and output (O), and the error modelling in between. Six levels are distinguished along this path. The model input can be vectorised information such as an object list (O) or rendering (R). The output is distinguished between object list (O), detections (D), or raw data (R). With these abbreviations, a naming scheme is provided from a combination of them: for example, object in object out (OIOO) or rendering in detection out (RIDO), and so on.

A new area of research in the modelling community combines the virtual world with real-world hardware components to drive a dedicated HiL testbed for radar sensor stimulation, because they provide the ability to thoroughly test radar sensors under laboratory conditions [45]. With advances in analog and/or digital millimetre-wave signal processing technology, powerful real-time radar target stimulators have emerged that can accurately stimulate the radar signature of real targets represented by point scatterers. The general operating principle is that the receiver mixes the received radar signal into the baseband, digitises the baseband signal, modifies the waveform on one or more FPGAs, and converts it into an analog signal. Possible signal modifications include amplitude and phase changes, time delays, and frequency shifts. Finally, the mixed radar echo is transmitted back over the air at the transmitter end. In [46], an HiL approach is used that emulates a virtual radar environment corresponding to a defined test scenario. The relevant test scenarios are parameterised and then mapped to the antennas of the target stimulator. A radar HiL test approach based on OTA stimulation can be found in [47,48]. In [47], a simulation platform is developed using multi-body simulation (MBS) software to combine traffic and scenario simulations. The radar echoes are mixed with Gaussian noise to improve the realism of the test. Based on the flexibility and scalability of available high-frequency hardware components combined with MBS, the authors presented a whole-vehicle-level DAS/ADS testbed based on OTA radar target stimulation in [49,50,51]. In [52], a dynamic OTA radar stimulator is demonstrated. The system provides the illuminated antennas with three degrees of freedom of motion to simulate more complex scenarios with different angular positions of the target vehicle. The authors in [53] calculate the radar characteristics using theoretical formulations and implement them in FPGA hardware components to produce even more realistic radar echoes. Another ViL test method based on the OTA radar target stimulation approach is presented in [54]. The ambient perception simulation is based on statistical distributions and the radar signature of the target is estimated as a function of the target vehicle dynamics. In addition to the aforementioned development and research systems, radar target stimulators are also used in series production as end-of-line (EOL) test equipment.

The open integration and test platform CarMaker of IPG Automotive GmbH classifies sensor models into three groups: Ideal, High Fidelity (HiFi) and Raw Signal Interface (RSI) [55]. Ideal sensor models represent a generic interface. Object information can be accessed within the defined sensor range, and the model is technology-independent. High-fidelity models correspond to phenomenological sensor models and provide a higher level of detail than ideal models. They use ideal environmental information and overlay it with technology-dependent effects known from theory and measurements. Physical models with an RSI account for actual signal propagation. This includes the main physical effects involving the interaction of the signal with objects in the simulation and the transmission media along the propagation path.

**Figure 3:**
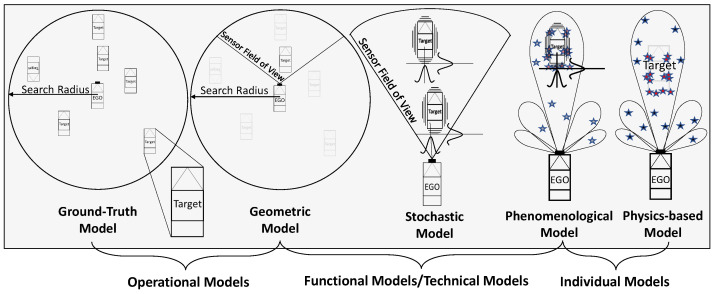
Sensor modelling approaches.

Furthermore, most simulation tools provide different interfaces for sensor modelling with varying complexity [55]. We used the definition given in [56], which describes the complexity of the model as follows:

The ground truth model contains all information about all objects within the search radius. The geometric models take into account some basic information from the sensor’s data sheet and map the detection area and field of view. The stochastic models assume some uncertainty in the detection probability and provide parallel measurements with artificially generated noise. The most complex models for perceptual sensors are physics-based models that use the physical properties of the object, wave propagation, and reflections. From the point of view of computational requirements and reduced parameter space, phenomenological models are a good alternative since mathematical methods can be used to simulate a sensor-specific phenomenon. This classification, presented in Figure 3, serves as the basis for a classification more focused on vehicle development applications, as described below.

## 5. Classification from the System Integrator’s Perspective

Modeling the performance of perceptual sensors at different levels of abstraction in the development process is critical because it provides a preliminary estimate of sensing capabilities, enabling the development and verification of different sensing strategies that are essential for automated driving functions [57]. The radar sensor models available in the literature have been studied extensively by researchers and, as described earlier, have been classified into many different categories.

X-in-the-loop test methods for verifying and validating the safety of ADSs range from field tests to simulation of all subcomponents of the complete vehicle. Due to the high complexity of an ADS, test activities are shifted to the virtual test domain by replacing one or more physical components with an appropriate simulation model [20,58]. The scenario-based method can be applied to the X-in-the-loop testing methodology throughout the development process described in Section 3 because the scenario description is based on different levels of abstraction or detail. Our motivation to introduce a new naming convention for the state-of-the-art radar sensor models is based on the scenario abstraction levels [59] and the X-in-the-loop test methods [60], which are jointly mapped to the phases of the development process represented in the V-model [61].

We have also defined two prerequisites: First, that the automotive manufacturer involves an DAS/ADS supplier with specialised knowledge and unique experience in the field of environmental perception sensing at the subsystem or at least component level. Second, radar sensor models with a more detailed output data level than object lists, such as target/cluster lists or raw signals, cannot be integrated into the vehicle development process by automotive manufacturers due to a lack of technological knowledge and hardware resources appropriate to high-frequency (77–79 GHz) automotive radar technology. It is assumed that these models are more likely to be used on the supplier side.

In increasing order of complexity, we introduce *operational models*, *functional models*, *technical models*, and *individual models*. The application of the different models in the vehicle development process is illustrated in Figure 2 and described below.

Following the V-model, the sequence of development phases is: *operational > functional > individual > technical > functional*. For OEMs, however, only the *operational, functional, technical,* and *functional* radar sensor models can be used. After applying the *technical models* in HiL testing at the component and sub-system level, *functional models* can be reused for DiL and ViL testing, for example, for testing the human–machine interface (HMI).

### 5.1. Operational Model

#### 5.1.1. Definition

The term operational means *“in or ready for use”*; ground truth and geometric models are considered. In the concept phase, simplified sensor models can be used to specify the perception concept of the automated driving system. For example, it must be determined which areas of the vehicle environment are to be perceived and at what distance objects must be detected. For this purpose, typical sensor properties such as sensor FOV, detection range, and so on can be modelled easily and quickly even without specific knowledge of perception sensor technology.

#### 5.1.2. Application

The automotive industry has a wide range of simulation tools to support the development process and to accelerate V&V test activities. In the concept phase, simplified sensor models can be used to specify the perception concept of the automated driving system. For example, it must be determined which areas of the vehicle environment are to be perceived and at what distance objects must be detected. In the further course of development, sensor models can support the selection of the sensor technology to be used for the automated driving system (radar, lidar, camera, etc.). For this purpose, typical sensor characteristics such as sensor FOV, detection range, and so on can be modelled. With the ability to generate the GT for each simulation step, all of these modern simulation tools have easy-to-parameterise, built-in, and technology-independent generic sensor models. These models are often referred to as idealised or geometric models and provide object lists as output data. As examples, we mention some products that are widely used in the automotive industry: in [62], TwT GmbH, TASS-PreScan, dSpace-ASM; in [57], TESIS Dyna4-Driver Assistance, MathWorks-ADAS Toolbox; in [63], CARLA, AirSim, DeepDrive, Udacity, Constellation, Helios, GLIDAR, RADSim, SIMSonic; and in [64], CarMaker from IPG Automotive GmbH, VIRES-VTD, CARLA, and AirSim can provide GT information.

In vehicle development, *operational models* are used in the descending branch of the V-model, in the early stage where the operational concept of the vehicle is tested with abstract scenarios against the requirements (see also Figure 2).

### 5.2. Functional Models

#### 5.2.1. Definition

The term functional means *"of or having a special activity, purpose, or task”.* Stochastic, phenomenological, and data-driven models are being considered with increasing attention. Given a particular sensor technology, such as the radar sensor, a functional representation of the acquisition process can be modelled by simulating the antenna properties with a simple cone and a complex target with a cuboid. Discretised scatterers can then be generated from the object, which can additionally be overlaid with noise to provide a more realistic object list. To accommodate different design considerations, the complexity can vary over a wide range. These models may require moderate sensor-specific knowledge to realise realistic behaviour with a reduced parameter space.

#### 5.2.2. Application

The output of a *functional model* usually does not deal with the internal processes or algorithms of the real sensor, but focuses on reproducing the effects that distinguish the sensor output from the reference data. Unlike *operational models*, *functional models* already contain more information and details about the real sensor properties. The authors in [27,36] illustrate this point that a non-parametric modelling approach is able to model sensor range, occlusion, latency, ghost objects, and object loss without explicit programming, and can be used efficiently in real-time simulation. The same concept is developed in [26,65], where the geometric information of the target is transformed into the sensor model, and then the signal noise and statistically based signal loss are superimposed on the original signal. The method described above has provided good estimation and modelling of relative distance, velocity, and other sensor-specific information. The data-driven approach requires a large number of experiments to obtain a statistical distribution that can be applied by the model. However, in the real world, there are often crucial parameters that affect the detection results, and a given statistical distribution may not do justice to the sensor’s detection performance. Therefore, a data-driven approach based on machine learning (ML) is introduced. In the work of [64], different ML methods are investigated and used to build RCS models, demonstrating that better prediction accuracy can be achieved with ML models. In addition to data-driven methods, geometric-based approaches are also commonly used for radar feature modelling. The geometric approach focuses more on the specific details of the target and models according to the statistics of the reflection points at different locations on the surface of the target to create the sensor-specific object list.

In vehicle development, *functional models* are used in the descending branch of the V-model after the use of *operational models*, but can also be reused in the ascending branch after the use of *technical models*. For more detailed logical scenarios, *functional models* can be used at the subsystem level in the design phase to produce sensor-technology-specific outputs that are used as inputs to a sensor fusion algorithm. In addition, the models can be used to verify that real sensors meet the requirements of the system specifications. In this regard, see also Figure 2. The figure shows that *functional models* can be used not only for verification purposes in the design phase, but also in the ascending branch of the V-model in the integration phase for testing. Since *functional models* are expected to perform their task in real time or even faster than real time, they can replace the real sensors for functional testing of the vehicle’s HMI in DiL testing or for testing some vehicle functions at the system level in ViL testing methods.

### 5.3. Technical Models

#### 5.3.1. Definition

*“Involving or concerned with applied and industrial sciences”*, models for over-the-air (OTA) target stimulator testbeds are considered. This group of radar sensor models includes models developed for a specific well-defined high-frequency radar target stimulator. These models have much lower performance compared to their simulation-only counterparts and are developed according to the available hardware components. These models typically provide only radial range, angular position, radial range rate, or velocity, as well as RCS information for radar signature generation in the form of a point target.

#### 5.3.2. Application

*Technical models* provide object lists with reduced content to represent a radar signature, usually in the form of a point target. The model output is the input data for a particular radar target stimulator high-frequency HiL testbed. A typical radar signature consists of: Doppler shift $f_{d}$ due to relative velocity, range in the form of propagation delay Δt, spatial direction (azimuth Φ, elevation Θ), and RCS σ describing the effective area of the identified objects. Once the concept of the perceptual sensor system is fully defined, the integration phase begins (right side of the V-model). For the integration of initial hardware prototypes with mostly limited functionality, *technical sensor models* provide input signals to validate the intended functionality on HiL, DiL, or even ViL test benches. Due to the lack of detailed technological knowledge of the subcomponents and the complex high frequency technology, the focus is on quantitative rather than qualitative or performance analysis.

Although OTA radar sensor simulation is widely used in co-simulation for HiL and ViL, the huge investment in hardware equipment is still a challenge. In addition, due to the high computational requirements of the real-time system, the effects of environmental conditions on the radar echo are often ignored or reduced to a probability distribution. Furthermore, the number of objects to be simulated is also limited.

In vehicle development, *technical models* are used in the ascending branch of the V-model, after the usage of *individual models*. The test cases are defined in concrete scenarios with well-defined requirements. *Technical models* support X-in-the-Loop on vehicle level, see also Figure 2.

### 5.4. Individual Model

#### 5.4.1. Definition

The term individual means *"single; separate, for a particular use”*; physically based models are considered here. Parametrisation is only possible with the expertise of the system supplier. To verify the detection performance of an individual sensor or a sensor cluster under non-optimal detection conditions, sensor-typical phenomena such as range reduction, multipath wave propagation, reflections, attenuation, or the unexpected rapid changes of the radar cross-section have to be modelled.

#### 5.4.2. Application

Under the *individual model*, physical models are considered, provided the sensor supplier has all the technology and hardware-specific parameters to perform a reliable performance evaluation. The application is in the lower part of the V-model, at the component or subsystem level, where performance verification can only be performed by the supplier. Models that provide raw data, reflection points and target lists belong to this class of models because the data processing algorithms for clustering and tracking are not known to the vehicle integrator. *Individual models* can be used effectively by system suppliers who have the technological knowledge to perform simulations of everything down to semiconductor components. Examples include ray-tracer-related models, any time-domain electromagnetic wave simulation, finite-difference time-domain (FDTD) method, finite element method (FEM), raw input-raw output (RIRO) method, raw input-object-output (RIOO) method, and so on [44].

Academic and industrial researchers are making great efforts to develop an efficient, easy-to-use sensor model that behaves like the real sensor. However, without the specific infrastructure, such as an echo chamber the size of a vehicle [66], or knowledge of characteristic parameters, such as the antenna radiation pattern [32], the modelling can only be based on compromises and must focus on a very specific domain or use case [67]. These compromises include simplifications and assumptions, for example:

- replacing of the real material description model by a probabilistic material model in [68];
- assuming the radiation pattern of the antenna is known in [32];
- replacing complex objects by multiple scattering centres in [69];
- or treating all metallic surfaces as perfect conductors (PEC) [54], while considering all other materials as absorbing in [40].

The authors show in [70] that a virtual representation of a real perceptual sensor in the form of a physics-based model is possible if the right supplier information and resources are available. In this paper, some basic properties of radar detection are investigated by measurement and calibrated simulation, including RCS estimation using ray tracer method. The main contribution of this work is that the calibration parameters for simulation can be derived from real measurements if the key parameters of the real sensor hardware are known.

In vehicle development, *individual models* are used in the ascending branch of the V-model, after the usage of *functional models*. The test cases are defined in concrete scenarios with well-defined requirements. *Individual models* support X-in-the-loop at the component and subsystem leve (see also Figure 2).

### 5.5. Classification Overview

Summarizing the results of our review in Section 4, we list existing approaches and review papers and the related classification approach in Table 1. Approaches can be found in the rows, and previous mentions in survey papers in columns.

We also organised the radar sensor modelling approaches found in the literature in our new classification scheme including operational, functional, technical, and individual models. For better readability, the whole table including the classification for each review paper is available at https://doi.org/10.3217/kgg17-wq710 (accessed on 24 June 2022 ). Here, the existing classifications from previous review papers are also mentioned.

The last row summarises how often the approach is mentioned in an existing review paper, which is an indicator of the recognition of the approach. A number zero means that previous reviews did not classify the approach.

## 6. Discussion

As complexity increases, the verification and validation (V&V) of automated driving systems (ADS) becomes exponentially more inefficient in terms of time-to-market and cost when the focus is on on-road testing. A variety of X-in-the-loop methods have been introduced to support efficient V&V of the safe performance of ADS. However, the quality of the predictions depends mainly on the ability of the V&V method to replicate the performance of real machine perception. Automotive radar sensors are superior in certain characteristics, such as performance in adverse weather conditions and precision in measuring the relative speed and distance of objects, especially moving objects. However, radar sensors are difficult to model due to the complex physical relationships between multipath propagation and electromagnetic wave reflection. In previous research, numerous attempts have been made to develop models using very different modelling techniques. In previous literature reviews, different classifications were established to structure the large amount of available research. This paper summarises the methods described in the literature, but also introduces a new perspective. Since X-in-the-loop-based approaches ultimately support the goal of whole-vehicle-level development, we have classified the available approaches in a perspective of how they are used in the development process and have introduced—in increasing order of complexity and use along the V-model integration approach— *operational models*, *functional models*, *technical models*, and *individual models*.

We summarised the different approaches and classifications and provided a comprehensive table that combines previous classifications with our new approach. Thus, the reader is able to quickly get an overview and select a suitable modelling method for further use. Finally, we provide a link to a dynamic spreadsheet that is publicly available at: https://doi.org/10.3217/kgg17-wq710. (accessed on 24 June 2022) This spreadsheet is being continuously enhanced as more progress in radar sensor modelling is achieved and will include comments of researchers and readers in that tabular overview in future.

## Figures and Tables

**Table 1:** Classification of radar sensor model approaches and relation to previous reviews.

		[23]	[25]	[71]	[30]	[19]	[41]	[43]	[42]	[44]	[67]	[18]	Sum:
OPERATIONAL	[72]								x	x			2
	[38]					x					x		2
	[73]					x				x		x	3
	[18]					x							1
	[74]									x	x		2
	[75]										x		1
	[28]		x										1
	[76]												0
	[77]	x											1
FUNCTIONAL	[27]		x			x				x		x	4
	[36]				x								1
	[26]	x	x			x				x			4
	[41]	x				x	x		x	x			5
	[39]												
	[65]												0
	[78]	x							x		x		3
	[79]												0
	[33]				x				x				2
	[64]												0
	[80]												0
	[81]									x			1
	[82]												0
	[83]								x				1
	[29]		x		x	x			x	x		x	6
	[84]												0
	[24]			x	x								2
	[71]	x											1
	[85]												0
	[86]												0
	[87]												0
	[37]				x				x				2
	[30]												0
	[88]				x								1
	[89]	x											1
	[90]							x					1
	[91]					x							1
	[57]							x					1
	[92]							x					1
	[93]									x			1
	[94]									x			1
	[95]									x			1
	[96]												0
	[97]												0
	[98]									x			1
	[99]	x		x									2
TECHNICAL	[45]												0
	[46]												0
	[47]												0
	[48]												0
	[49]												0
	[50]									x			1
	[100]												0
	[51]												0
	[52]												0
	[53]											x	1
	[101]									x			1
	[102]									x			1
	[103]												0
	[54]					x				x			2
FUNCTIONAL	[69]	x											1
	[32]			x	x					x	x	x	5
	[68]												0
	[67]									x			1
	[70]												0
	[104]					x							1
	[105]												0
	[35]				x	x		x		x			4
	[106]									x		x	2
	[40]					x					x		2
	[107]												0
	[108]												0
	[109]	x											1
	[110]	x				x							2
	[111]									x			1
	[112]											x	1
	[31]				x								1
	[34]				x								1
	[113]					x							1
	[114]					x				x			2
	[115]					x							1
	[116]					x							1
	[117]					x							1
	[118]							x		x			2
	[119]								x				1
	[120]									x			1
	[121]								x				1
	[122]								x				1
	[123]								x				1
	[124]								x		x		2
	[125]								x				1
	[126]									x			1
	[127]									x			1
	[128]									x			1
	[129]									x			1
	[130]									x			1
	[55]									x			1
	[131]									x			1
	[132]										x		1
	[133]												0
	[134]												0
	[135]												0
	[136]	x											1

## Data Availability

The full classification data, presented in a comprehensive tabular format can be found at: the link has been changed to the same link as in the discussion section https://doi.org/10.3217/kgg17-wq710 (accessed on 24 June 2022). In addition, the literature cited in this table is provided in END-NOTE format.

## Abbreviations

The following abbreviations are used in this manuscript:

ACC	Adaptive Cruise Control
ADF	Automated Driving Function
ADS	Automated Driving Systems
AEB	Autonomous Emergency Braking
AEBS	Advanced Emergency Braking System
ALKS	Automated Lane Keeping Systems
C-NCAP	Chinese New Car Assessment Program
DAS	Driving Automation System
DDM	Data-Driven Model
DiL	Driver-In-the-Loop
E/E	Electrical and/or Electronic
EM	Electro Magnetic
EOL	End-Of-Line
Euro NCAP	European New Car Assessment Program
FDTD	Finite Difference Time Domain
FEM	Finite Element Method
FOT	Field Operational Test
FOV	Field of View
FPGA	Field Programmable Gate Array
GT	Ground Truth
HiFi	High Fidelity
HiL	Hardware-In-the-Loop
HMI	Human-Machine Interface
IRM	Ideal Radar Model
ISO	International Organization for Standardization
JNCAP	Japanese New Car Assessment Program
LCDAS	Lane Change Decision Aid System
LDW	Lane Departure Warning
LIDAR	Light Detection and Ranging
LKAS	Lane Keeping Assistance System
MBS	Multi-Body Simulation
MBSE	Model-Based Systems Engineering
MiL	Model-In-the-Loop
ML	Machine Learning
NCAP	New Car Assessment Program
NHTSA	National Highway Traffic Safety Administration
OEM	Original Equipment Manufacturer
OIOO	Object In Object Out
OTA	Over-The-Air
PALS	Partially Automated Lane Change System
PEC	Perfect Conductors
PiL	Processor-In-the-Loop
RADAR	Radio Detection and Ranging
RCS	Radar Cross Section
RIDO	Rendering In Detection Out
RIOO	Raw Input Object Output
RIRO	Raw Input Raw output
RSI	Raw Signal Interface
RTM	Ray-Tracing based Model
SAE	Society of Automotive Engineers
SC	Scattering Centre
SiL	Software-In-the-Loop
SOA	State-Of-the-Art
SOTIF	Safety Of The Intended Function
UN-ECE	United Nations Economic Commission for Europe
V&V	Validation and Verification
ViL	Vehicle-In-the-Loop
